# Supplementary material for: Content-rich biological network constructed by mining PubMed abstracts
Source: BMC Bioinformatics. 2004 Oct 8;5:147. doi: 10.1186/1471-2105-5-147 (PMC528731; doi:10.1186/1471-2105-5-147)
Supplement: Additional File 2 — The original results of the above study (non-essential files are deleted to keep the file size under the limit set by BMC bioinformatics). [file 1471-2105-5-147-S2.bz2 › chilibotAdditionalFile2/dip30/224ID7831290E740/html/PLCG1_SYK.html]

 


 **PLCG1** and **SYK** 
  
Found 77 abstracts in PubMed, retrieved 30.  
 

 What does Google say? 
 PDF only 
| .edu only 

---

**Interactive relationship** (e.g. stimulation, inhibition, etc)

**Neutral relationship**- FcepsilonRI ligation is able to induce major signaling events like protein tyrosine kinase activation including p72  **syk**  leading to PLC gamma1  [ **PLCG1** ]  phosphorylation and consecutive calcium influx.  Ref: 11306920 Int Arch Allergy Immunol< MedlineTA>Int Arch Allergy Immunol,
- Importantly, CD3 induced responses, such as tyrosine phosphorylation of cellular substrates LAT, SLP76, and PLC gamma1  [ **PLCG1** ] , as well as calcium mobilization, which are defective in T cells expressing neither ZAP 70 nor  **Syk** , are observed in  **Syk**  hi ZAP 70 T cells.  Ref: 10748099 J Biol Chem, 2000
- H 2 O 2 induced p38 activation is abrogated in phospholipase C gamma2 PLC  [ **PLCG1** ]  gamma2 deficient as well as  **Syk**  deficient cells, suggesting that  **Syk**  activates p38 through PLC  [ **PLCG1** ]  gamma2 upon H 2 O 2 stimulation.  Ref: 12215219 Antioxid Redox Signal, 2002
- These data indicate that the C terminal SH2 domain of PLC  [ **PLCG1** ]  gamma2 is important for PLC  [ **PLCG1** ]  gamma2 regulation through possible interactions with SLP 76,  **Syk** , Lyn, LAT and the FcR gamma chain.  Ref: 10469124 Eur J Biochem, 1999
- Until recently, the molecular mechanism underlying the double requirement for  **Syk**  and Btk in PLC  [ **PLCG1** ]  gamma2 activation remained unclear, but new data suggest that an adapter molecule, B cell linker protein alternatively named SLP 65 or BASH, phosphorylated by  **Syk** , provides docking sites for Btk SH2 domain as well as PLC  [ **PLCG1** ]  gamma2 SH2 domains, thus bringing Btk into close proximity with PLC  [ **PLCG1** ]  gamma2.  Ref: 11043765 Immunol Rev, 2000
- In oxidative stress, the C terminal SH2 domain of  **Syk**  was demonstrated to be required for induction of tyrosine phosphorylation of cellular proteins, phospholipase C PLC  [ **PLCG1** ]  gamma2 phosphorylation, inositol, 5 triphosphate IP 3 generation, Ca 2 release from intracellular stores, and c Jun N terminal kinase activation.  Ref: 10788787 J Biochem Tokyo , 2000
- Here, based upon the available genetic and biochemical data of the BCR signal components, we discuss several mechanisms by which BCR signals are propagated and modified, with specific emphasis on the phospholipase C PLC  [ **PLCG1** ]  gamma2 calcium pathway Gene targeting experiments in DT40 chicken B cells highlighted the importance of the intracellular protein tyrosine kinases  **Syk**  and Btk in PLC  [ **PLCG1** ]  gamma2 activation.  Ref: 11043765 Immunol Rev, 2000
- Finally, in cells deficient in the PLC  [ **PLCG1** ]  gamma2, one of the phosphorylated substrates regulated by  **Syk**  and Btk, TPA induced PLD activation, as well as phosphatidylinositol bisphosphate PIP2 hydrolysis was remarkably reduced.  Ref: 11380624 Genes Cells, 2001
- We also show that Tec family PTKs enhanced endogenous phospholipase C PLC gamma1  [ **PLCG1** ]  phosphorylation induced by CD8 zeta and  **Syk**  in 293T cells.  Ref: 11240141 FEBS Lett, 2001
- Activation of src and  **Syk**  kinases plays a significant role in signal transduction of heterophil degranulation probably by stimulating downstream phosphorylation of PLC  [ **PLCG1** ] , PLD, and PI3 K.  Ref: 12757738 Int Immunopharmacol, 2003

**Non-interactive relationship** (e.g. studied together, co-existance, homology, etc.)

- Inhibition of the tyrosine kinase  **Syk**  by piceatannol does not affect vWF induced tyrosine phosphorylation of Fc gamma RIIA but prevents phosphorylation of PLC  [ **PLCG1** ]  gamma 2.  Ref: 11344169 J Biol Chem, 2001
- These were found to include PLC  [ **PLCG1** ]  gamma2,  **Syk** , SH2 domain containing leucocyte protein of 76 kDa SLP 76, Lyn, linker for activation of T cells LAT and the FcR gamma chain.  Ref: 10469124 Eur J Biochem, 1999
- In B cells from Cbl b deficient mice cross linking the BCRs resulted in sustained phosphorylation of Igalpha,  **Syk** , and phospholipase C PLC  [ **PLCG1** ]  gamma2, leading to prolonged Ca2 mobilization, and increases in extracellular signal regulated kinase ERK and c Jun NH2 terminal protein kinase JNK phosphorylation and surface expression of the activation marker, CD69.  Ref: 12771181 J Exp Med< MedlineTA>J Exp Med, 1999
- Coligation of the cytoplasmic region of PD 1 with the B cell receptor BCR in IIA1.6 transformants inhibited BCR mediated growth retardation, Ca 2 mobilization, and tyrosine phosphorylation of effector molecules, including Ig beta,  **Syk** , phospholipase C gamma 2 PLC  [ **PLCG1** ]  gamma 2, and ERK1 2.  Ref: 11698646 Proc Natl Acad Sci U S A, 2001
- These findings have led to the suggestion that BLNK is required for coupling  **Syk**  to PLC  [ **PLCG1** ]  gamma2, thereby accelerating cell apoptosis in B cells exposed to low doses of H2O2.  Ref: 11813980 Antioxid Redox Signal, 2001
- **Syk** , linker for activated T cells, and phospholipase C gamma 2 PLC  [ **PLCG1** ]  gamma 2 are among the proteins tyrosine phosphorylated on convulxin stimulation in PMA differentiated HEL cells.  Ref: 11023507 Blood, 2000
- Whereas tyrosine phosphorylation of Fc epsilon RI,  **Syk** , and Vav was intact in LAT deficient BMMCs following Fc epsilon RI engagement, tyrosine phosphorylation of SLP 76, PLC gamma1  [ **PLCG1** ] , and PLC  [ **PLCG1** ]  gamma2 and calcium mobilization were dramatically reduced.  Ref: 10843385 Immunity, 2000
- The enhanced phosphorylation of mSH2 N  **Syk**  and PLC  [ **PLCG1** ]  gamma2 did not link to Ca 2 mobilization from intracellular pools and IP 3 generation.  Ref: 10788787 J Biochem Tokyo , 2000
- Endogenous B cell receptors BCR coupled through  **Syk**  kinase to phospholipase C gamma PLC  [ **PLCG1** ]  gamma activated the expressed TRPC3 channels in both DT40w t and DT40InsP 3 R k o cells.  Ref: 11466302 J Biol Chem, 2001
- Expression of SH2 domain containing leukocyte specific phosphoprotein of 76 kDa SLP 76, a hematopoietic cell specific adapter protein, is required to couple  **Syk**  family tyrosine kinase activation to downstream mediators such as phospholipase C PLC  [ **PLCG1** ]  gamma following TCR, platelet collagen receptor and mast cell Fc epsilon R stimulation.  Ref: 10837416 Int Immunol, 2000
- Calcium mobilization in BLNK deficient as well as  **Syk**  deficient and phospholipase C PLC  [ **PLCG1** ]  gamma2 deficient cells after H2O2 treatment was completely abolished.  Ref: 11813980 Antioxid Redox Signal, 2001
- Tyrosine phosphorylation of phospholipase C gamma1  [ **PLCG1** ]  but not of  **Syk**  and calcium mobilization in response to IgE cross linking were reduced in SLP 76 deficient BMMCs.  Ref: 10377180 J Clin Invest, 1999
- In contrast, the tumor cells in all cases of lymphocyte predominance Hodgkin rad s disease were positive for Fyn,  **Syk** , BLNK and PLC  [ **PLCG1** ]  gamma2, and Lyn immunostaining was seen in a minority of biopsies.  Ref: 12881301 BloodBlood, 1999
- Similarly, vWF induced tyrosine phosphorylation of Fc gamma RIIA, as well as of  **Syk**  and PLC  [ **PLCG1** ]  gamma 2, occurs normally in aspirinized platelets.  Ref: 11344169 J Biol Chem, 2001
- Fc varepsilon RI induced tyrosine phosphorylation of phospholipase C gamma PLC  [ **PLCG1** ]  gamma but not  **Syk**  was significantly suppressed in these cells.  Ref: 12810085 Biochem Biophys Res Commun, 2003
- The Src family kinase  **Syk** , the B cell adaptor protein BLNK, and phospholipase PLC  [ **PLCG1** ]  gamma2 were consistently absent from Reed Sternberg cells.  Ref: 12881301 BloodBlood, 2003
- CONCLUSIONS We demonstrated that the  **Syk** , Btk and PLC  [ **PLCG1** ]  gamma2 pathways are required for TPA induced PLD activation in DT40 cells.  Ref: 11380624 Genes Cells, 2001
- Platelet activation by collagen is mediated by the sequential tyrosine phosphorylation of the Fc receptor gamma chain FcR gamma chain, which is part of the collagen receptor glycoprotein VI, the tyrosine kinase  **Syk**  and phospholipase C gamma2 PLC  [ **PLCG1** ]  gamma2.  Ref: 10469124 Eur J Biochem, 1999
- In contrast, in mSH2 N  **Syk**  expressing cells, tyrosine phosphorylation of intracellular proteins including PLC  [ **PLCG1** ]  gamma2 was markedly induced in oxidative stress.  Ref: 10788787 J Biochem Tokyo , 2000
- Engaging CRs on chicken heterophils activates a proximal tyrosine kinase src and  **Syk**  kinases >PLC  [ **PLCG1** ]  PLD >PI3 K >JNK signal transduction pathway that induces degranulation.  Ref: 12757738 Int Immunopharmacol, 2003
- WGA, which is a very potent platelet stimulator, elicited a rapid surge in  **Syk**  and phospholipase C PLC  [ **PLCG1** ]  gamma 2 tyrosine phosphorylation and the resultant intracellular Ca 2 mobilization.  Ref: 11669637 Biochemistry, 2001
- The absence of an in vivo association between  **Syk**  and PLC  [ **PLCG1** ]  gamma2 in platelets is in contrast with that for PLC gamma1  [ **PLCG1** ]  and  **Syk**  in B cells.  Ref: 10469124 Eur J Biochem, 1999
- Engaging Fc receptors on chicken heterophils activates a  **Syk**  >PLC  [ **PLCG1** ]  >PI3 K >p38 MAPK signal transduction pathway that induces degranulation.  Ref: 12188037 Int Immunopharmacol, 2002
